# Marker Assisted Selection (MAS) for chickpea *Fusarium oxysporum* wilt resistant genotypes using PCR based molecular markers

**DOI:** 10.1007/s11033-014-3561-3

**Published:** 2014-07-14

**Authors:** Zakia Ahmad, Abdul Samad Mumtaz, Abdul Ghafoor, Amjad Ali, Mohammad Nisar

**Affiliations:** 1 Department of Botany, University of Malakand, Chakdara Dir (Lower), Khyber Pakhtunkhawa Pakistan; 2 Department of Plant Sciences, Quaid-i-Azam University, Islamabad, Pakistan; 3 Principal Scientific Office, IABGR, NARC, Islamabad, Pakistan; 4 Department of Biotechnology, University of Malakand, Chakdara Dir (Lower), Khyber Pakhtunkhawa Pakistan

**Keywords:** Chickpea, Wilt disease, Molecular markers, ROC, Curve analysis

## Abstract

The exploration of genetically superior accessions is the key source of germplasm conservation and potential breeding material for the future. To meet the demand of better yielding chickpea cultivars in Pakistan the present study was organized to select more stable and resistant lines from indigenous as well as exotic chickpea germplasm obtained from Plant Genetic Resource Institute (PGRI), National Agricultural Research Centre, Islamabad, Pakistan. For the identification and evaluation of chickpea wilt resistant lines against *Fusarium oxysporum* f. sp. *ciceris* (Schlechtends), the germplasm was tested in the field for the selection of wilt resistant lines and the PCR based molecular markers were investigated to use Marker Assisted Selection (MAS) for selection of the desirable cultivars. In field trial, 70 % accessions were resistant to wilt disease, while the remaining 30 % have shown susceptibility to the disease. A total of 5 RAPD and 15 SSR markers were screened for molecular based characterization of wilt response. The data of molecular markers were scored by the presence (1) and absence (0) of allele and subjected to statistical analysis. The analysis was based on coefficient of molecular similarity using UPGMA and sorted the germplasm into two groups based on disease response. Among the total used RAPD/SSR primers, only TA194 SSR marker showed linkage to wilt resistant locus at 85 % probability. The linkage of a marker was reconfirmed by receiver operating characteristic curve. The use of the sorted wilt resistant genotypes through SSR marker TA194 can make available ample prospect in MAS breeding for yield improvement of the crop in Pakistan.

## Background

 Chickpea (*Cicer arietinum* L.) is an important food legume and a protein rich cash crop has been classified into two main types; small dark-seeded Desi type of Indian origin and large light-seeded Kabuli type of Mediterranean origin [1]. Pakistan is the major grower country of chickpea in the world, where it is cultivated on about one million hectares with a total production of 760 thousand tons [2]. While, in Khyber Pakhtunkhwa it was cultivated on an area of 42 thousand hectares with 20 thousand tons annual production [3]. Although it is grown on large area, but the main reasons of its very low yield and production are either biotic/abiotic stresses, selection strategies for development of desirable traits cultivars and poor labour management [4–8]. In addition among various environmental constraints, one of the limiting factors which directly affected the yield and causing 10–90 % loss to the crop [9], is the fungal disease caused by *Fusarium oxysporum* sp. *ciceris* (Schlechtends) which causes chickpea wilting. Chickpea wilt is gradually prevailing in Pakistan as a result of the increased drought condition since last few years. Therefore, the issue needs great attention to enhance the yield [10]. The disease is soil or seed born [11], which is difficult to control by the use of chemicals or fungicides [12]. To overcome this serious problem, the use of resistant and quality cultivars to control wilt is the best and cheapest way for breeders to adopt [13]. In Pakistan there does exist a wide gap between its potential and real yield attributed by different constraints; unfortunately in traditional farming system the farmers still in use of old chickpea cultivars and varieties due to unavailability of the attainments of chickpea upgrading research programs to increase the yield of a crop at homestead level. However for substantial increase in the efficiency of chickpea production which is the requirement of developing countries like Pakistan to overcome on food problems, needed to adopt the use of quality seeds with allied scientific technologies by the chickpea growers. Chickpea production in the country can be stabilized and improved by the development of suitable chickpea cultivars adaptable for all sorts of environments [14]. The selection and inheritance of the desirable traits is now become possible with the advancement of Marker Assisted Selection (MAS) which provides a beneficial source to exploit the potentiality of genes against agronomic traits [15, 16]. In this connection a set of PCR based currently available RAPD and SSR markers are often chosen for their higher genome coverage [17, 18]. In previous studies the linkage map of resistance genes for *FOC* 1–5 races was developed using different RAPD and SSR markers in recombinant inbred lines (RILS) populations generated from various resistant and susceptible parental combinations [19–22]. While at least eight races of this fungus have been reported, out of which six are more virulent causing wilt disease [23, 24]. However, there is no any information about the existence of races in Pakistan. It has also been reported by many workers [25–28], that virulent races of the pathogens need continuous characterization for screening of germplasm because of constantly changing their nature after some time from resistant to susceptible. In addition, the conventional pathotyping techniques are no more valid for reliable evaluation and identification of wilt causing fungal pathogens [29]. Therefore, the present study was organized to select the resistant and susceptible lines in unreported chickpea local (Pakistani) and exotic (USA) germplasm through a set of RAPD and SSR markers linkage to resistance genes for future resistance gene pyramiding and to enhance resistant germplasm resources for increasing yield of chickpea in Pakistan.

## Materials and methods

### Plant materials

Twenty-four indigenous and 46 exotic accessions of chickpea were obtained from Plant Genetic Resource Institute (PGRI), National Agriculture Research Centre, Islamabad, Pakistan (Catalogue) for field experiments performed in the research area of Malakand University, Chakdara, Khyber Pakhtunkhwa, Pakistan during 2009–2012 [30]. For planting the accessions, randomized complete block design (RCBD) suggested by Clewer and Scarisbrick [31] was used, keeping row to row distance 75 cm with row length of 5 m.

### Disease screening

Chickpea germplasm was tested for wilt resistance in field against *F. oxysporum* f. sp. *ciceris* (*FOC*) using the isolates provided by the Department of Pathology, University of the Punjab, Pakistan. The fungal inoculum was increased by multiplying with sorghum grains. At the time of inoculation, each of the test isolate was mixed thoroughly to develop wilt sick bed, where the accessions were plotted in rows.

### RAPD/SSR molecular markers

For Molecular characterization genetic linkages both RAPD and SSR primers were screened (Tables 1, 2). Five RAPD and 15 SSR primers were tested for genetic linkage. The DNA was extracted from dry seeds through a modified technique of Kang et al. [32]. Whereas, quality of the genomic DNA was ensured through agarose gel electrophoresis. The quantification was done through Spectrophotometer with accordance to the instructions provided in the literature of the instrument protocol booklet.

**Table 1:** Sequences of the RAPD primers used in the present study for molecular analysis of chickpea germplasm

S/no.	Primer name	Sequence (5′–3′)
1	UBC 181	ATGACGACGG
2	UBC 733b	GGGAAGGGAG
3	OPA4	AATCGGGCTG
4	OPA9	GGGTAACGCC
5	OPG13	CTCTCCGCCA

**Table 2:** Sequences of the SSR primers used in the present study for molecular analysis of chickpea germplasm

S/no.	Primer name	Sequence forward/reverse	No. of bands	Molecular weight (bp)
1	CaSTMS2	ATTTTACTTTACTACTTTTTTCCTTTC AATAAATGGAGTGTAAATTTCATGTA	2	114
2	CaSTMS15	CTTGTGAATTCATATTTACTTATAGAT ATCCGTAATTTAAGGTAGGTTAAAATA	1	159
3	CaSTMS21	CTACAGTCTTTTGTTCTTCTAGCTT ATATTTTTTAAGAGGCTTTTGGTAG	1	60
4	TA72	GAAAGATTTAAAAGATTTTCCACGTTA TTAGAAGCATATTGTTGGGATAAGAGT	1	198
5	TA130	TCTTTCTTTGCTTCCAATGT GTAAATCCCACGAGAAATCAA	1	219
6	TA194	TTTTTGGCTTATTAGACTGACTT TTGCCATAAAATACAAAATCC	2–3	204
7	TA71	CGATTTAACACAAAACACAAA CCTATCCATTGTCATCTCGT	1	202
8	TA22	TCTCCAACCCTTTAGATTGA TCGTGTTTACTGAATGTGGA	1	228
9	TA200	TTTCTCCTCTACTATTATGATCACCAG TTGAGAGGGTTAGAACTCATTATGTTT	1	296
10	TA46	TTTATTGCAATAAAACTCATTTCTTATC TTCTTTTTGTGTGAAAAAAAAATATAGTA	1	239
11	TA135	TGGTTGGAAATTGATGTTTT GTGGTGTGAGCATAATTCAA	1	192
12	TR1	CGTATGATTTTGCCGTCTAT ACCTCAAGTTCTCCGAAGT	1	224
13	TR7	GCATTATTCACCATTTGGAT TGTGATAATTTTCTAAGTGTTTT	1	204
14	TR29	GCCCACTGAAAAATAAAAAG ATTTGAACCTCAAGTTCTCG	2	220
15	TR31	CTTAATCGCACATTTACTCTAAAATCA ATCCATTAAAACACGGTTACCTATAA	1	217

### PCR amplification

To optimize the conditions for polymerase chain reaction (PCR) 25 µl of reaction mixture was prepared. For PCR reproducibility 2× concentrated solution of PCR master mixture (0.05 µl *Taq* DNA polymerase, Reaction buffer, 4 mM MgCl2 and 0.4 mM of each dNTP) was used in the reaction. Thermal cycling was optimized with denaturation temperature for 2 min at 94 °C, annealing temperature for 1 min at 55 °C and extension temperature 72 °C for 10 min. The PCR product was resolved on 2 % agarose gel in 1 × TBE buffer at 100 V. Tracking dye was mixed in PCR tube (containing mastermix) and short spinned to mix well. The PCR product was run and visualized the DNA profile under gel documentation system for the scoring of data for linkage analysis.

### Data analysis

The observations were made in rates (%) of accessions showed wilting at seedling stage, flowering time and complete response till pods maturity by using the wilt incidence formula [33].

$$({\text{Wilt}} \; {\text{incidence}} \,(\% ) = \frac{{{\text{Number}}\,{\text{of}}\,{\text{wilted}}\,{\text{plants}}}}{{{\text{Total}}\,{\text{number}}\,{\text{of}}\,{\text{plants}}}} \times 100)$$

The degree of susceptibility and resistance to disease of each line was determined by using 1–9 rating scale given by [34], which scored = 1 for highly resistant, resistant = 3, moderately resistant = 5; susceptible = 7, and highly susceptible = 9. The data from electrophorogram was scored by the presence (1) and absence (0) of allele. The variation intensity was not taken in consideration, but the linkage of molecular marker with wilt was scored. On the basis of presence and absence of alleles (bands), cluster analysis of 70 lines was performed to sort the lines with response to disease status. Coefficient of similarity based on UPGMA was performed. For Pearson correlation *t* test (alpha ≤ 0.05) was applied using STATISTICA version 7 for Windows. The probability of molecular marker was estimated and confirmed through receiver operating characteristic (ROC) curve analysis.

## Results

**Fig. 1:**
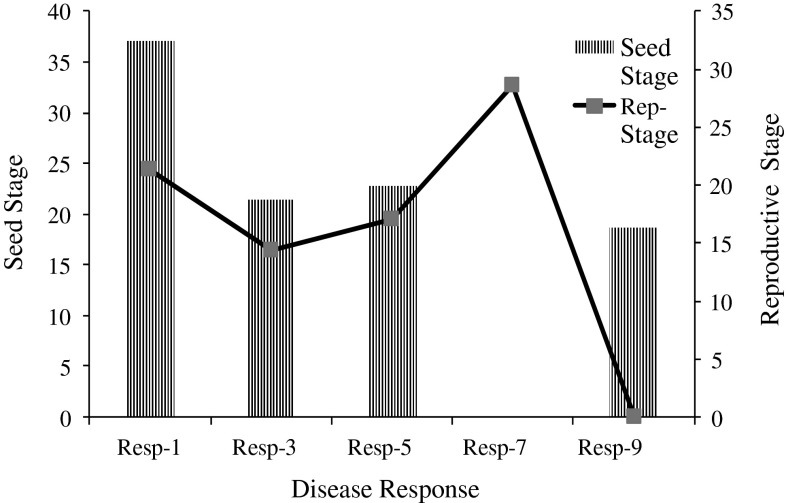
Wilt incidence (%) of chickpea 70 genotypes for *Fusarium* wilt disease response. *Resp* = response to disease, *1* = higher resistance (HR); *3* = resistant (R); *5* = moderate resistance (MR); *7* = susceptible at reproductive stage (SR); *9* = susceptible at seedling stage (SS)

In field screening 70 % accessions were observed as resistant and 30 % found susceptible to *Fusarium* wilt (Table 3). According to disease rating scale the total germplasm was categorized into highly resistant (HR), with wilt incidence (%) 37.1, resistant (R), with wilt incidence (%) 21.4, moderately resistant or tolerant (MR) with 22.8 wilt incidence (%) and highly susceptible (SS) group for which wilt incidence (%) was 18.6 at seedling stage. On the other hand the disease response of both local and exotic accessions at reproductive to pod maturity stage, scored HR, R, moderately resistant (MR) and susceptible (SR) lines with wilt incidence (%) calculated as 21.4, 14.3, 17.1 and 28.6 % respectively (Table 4; Fig. 1). Thus the average value of wilt incidence of the resistant group (HR, R and MR) at seedling stage was 27.1 % that dropped to 17.6 % at reproductive stage. Contrary to this, the susceptible group (SS and SR) raised at reproductive to maturity stage from 18.6 to 28.6 % respectively. Results regarding resistance to wilt disease of chickpea lines at both seedling and pods maturity stage presented in the Table 5 showed significant and distinct variation at alpha ≤ 0.050.

**Table 3:** Field screening data of chickpea 70 accessions against *Fusarium* wilt disease

Accessions distributed with reference to disease response	No. of acc. contributed	Percent contribution	1–9 Rating scale score	Disease response
1898, 2023, 2188, 2235, 2236, 2430, 2441, 2553, 2562, 2595, 2611, 3037, 3039, 3043, 3054, 3056, 2819, 2831, 3059, 2855	20	28.57	1	Highly resistant
2272, 2273, 2473, 2499, 2531, 2558, 2654, 3011, 2532, 3020, 3021, 3023, 3035, 3041, 3045, 3046, 3057, 3065, 3066, 3063	20	28.57	3	Resistant
1995, 1998, 3015, 3032, 3042, 3026, 3024, 3058, 3061	9	12.86	5	Moderately resistant
3027, 3031, 3033, 3040, 3044, 3047, 2629, 2650, 2859, 3062, 3064, 2544	12	17.14	7	Susceptible
2234, 1936, 2237, 2278, 2497, 3022, 3017, 3016, 2616	9	12.86	9	Highly susceptible

**Table 4:** Wilt incidence (%) of 70 chickpea accessions against *Fusarium* wilt disease at Seedling and reproductive to pods maturity stage

Disease response (1–9 rating scale)	Seedling stage wilt incidence (%)	Reproductive to pods maturity stage wilt incidence (%)
1. HR	37.1	21.4
3. R	21.4	14.3
5. MR	22.8	17.1
Ave. resistance response	27.1	17.6
7. SR	–	28.6
9. SS	18.6	–

*Ave* average, *HR* represent highly resistance genotypes (1–9 rating scale score = 1), *R* resistance (1–9 rating scale score = 3), *MR* moderately resistance (1–9 rating scale score = 5), *SR* susceptible (1–9 rating scale score = 7), *SS* highly susceptible (1–9 rating scale score = 9)

**Table 5:** *t*-Test for *Fusarium* wilt response of chickpea local and exotic lines

SOV	*t*-value	df	Mean	Mean			CI 95 %		
				df	SE	SD	Lower	Upper	*P* value
Seedling stage	6.032	3	17.5	17.5	2.901	5.802	8.267	26.73	0.01
Reproductive/pods maturity stage	6.553	3	14.25	14.25	2.175	4.349	7.329	21.17	0.01

Alpha ≤ 0.050

*df* difference, *SE* standard error, *SD* standard deviation, *CI* confidence interval

### Linkage of molecular markers

**Fig. 2:**
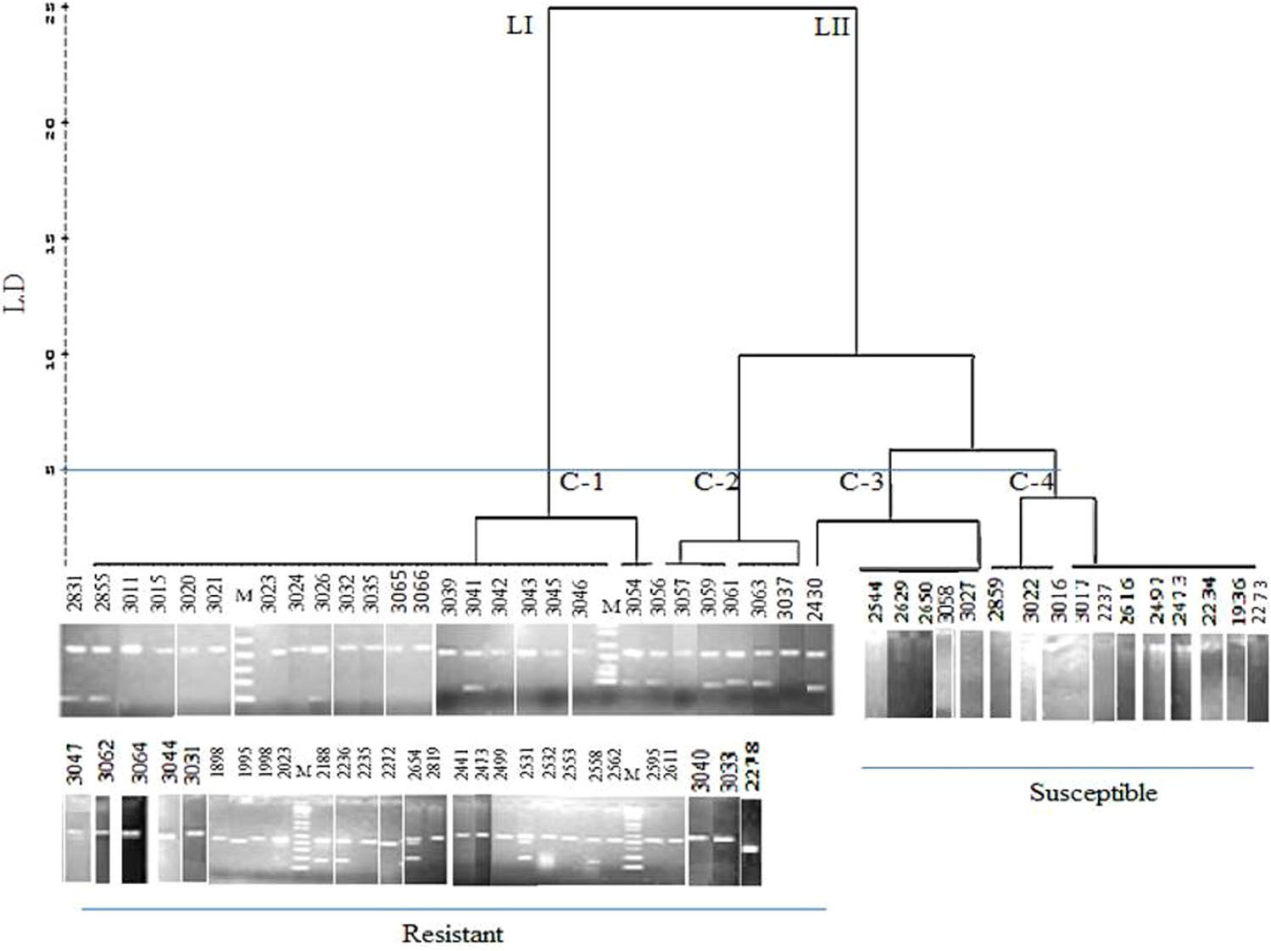
Comparative picture of field screening and PCR data for delimitation of resistant and susceptible accessions of chickpea germplasm

**Fig. 3:**
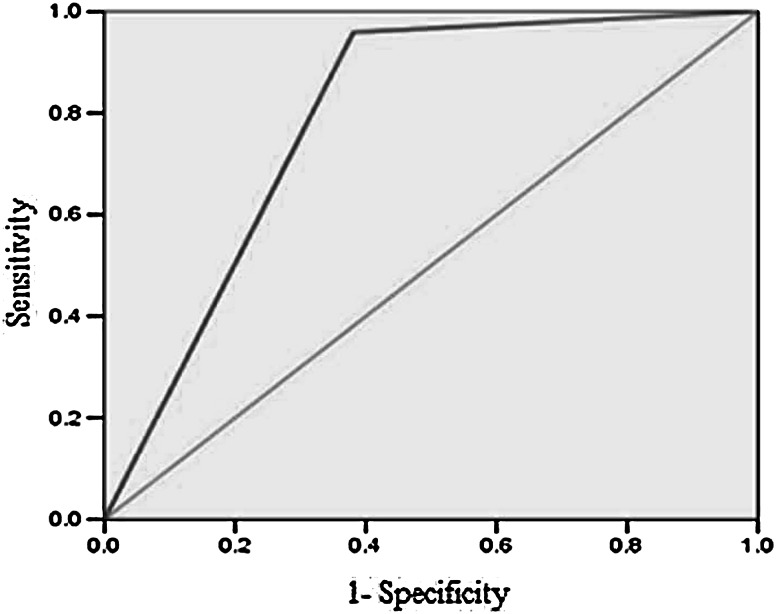
Receiver operating characteristic (ROC) curve to show range of resistivity against *Fusarium* wilt in the presence of resistant gene in chickpea accessions

To further evaluate and identified wilt resistance lines among chickpea germplasm, five RAPD and fifteen SSR markers were investigated to assess linkage with *Fusarium* wilt resistance gene. These primers were selected from previous literature [35, 36]. However in present study the SSR marker TA194 has only shown significant relation with the presence of allele for resistance (Table 6); therefore, it has been selected for further analysis. The dendrogram constructed on the basis of coefficient of similarity using UPGMA divided the total germplasm into two lineages and four clusters resulted in splitting of 70 accessions into two groups. The first group displayed 77 % accessions resistant to wilt disease, while the remaining 23 % grouped as susceptible (Fig. 2). The linkage probability of TA194 marker was 85 % (Table 7), and the association of the marker was reconfirmed by ROC curve (Fig. 3). The coefficient of correlation of marker TA194 with disease resistant gene (*FOC* locus), Factor 1 was highly significant at *P* ≥ 0.01 (Table 6). The PCR amplification using TA194 however; for certain accessions have shown multiple bands (Fig. 2).

**Table 6:** Coefficients of correlation between resistance and allele

	Estimate	Std. error	z value	Pr (>|z|)
Intercept	−1.8718	0.7596	−2.464	0.0137*
Factor (allele) 1	3.6425	0.8504	4.283	1.84e−05***

Significant codes: 0 ‘***’ 0.001 ‘**’ 0.01 ‘*’ 0.05 ‘.’ 0.1 ‘’ 1

Factor 1—band present in wilt resistant lines

**Table 7:** Association of level of probability of resistance with presence of allele

	a = −1.8718		
	b (allele 1) = 3.6425		
Probability of resistance when allele is present	p = e^(a + b)^/1 + e^(a + b)^	0.854684	0.854
Probability of resistance when allele is absent	p = e^a^/1 + e^a^	0.133197	0.1333

## Discussion

The MAS enhance sources of distinction and make easy the complex traits selection that is otherwise time consuming process when evaluated phenotypically. The procedure of MAS for disease resistance which is typically a quantitative trait can be more efficiently developed [37]. The stability among various genotypes to select high yielding and disease free chickpea lines is the key criterion for future breeding programs. A high level of resistance in chickpea genotypes against *Fusarium* wilt disease has been studied [38–42]. But identification and evaluation of chickpea wilt resistant lines against *F. oxysporum* f. sp. *ciceris* aiming at to combine field screening linked with gene using PCR based markers is a new avenue in chickpea breeding in Pakistan.

The germplasm categorized on the basis of disease response at seedling and reproductive stage for comparison provided a valid conclusion and this increase in susceptibility to wilt disease was observed that may be due to slow wilting resistance of certain chickpea accessions required long time for wilting. The *t*-test however, indicated that chickpea both from indigenous and exotic origin showed significant variation at alpha ≤ 0.050 at seedling and reproductive stage; has already been reported [43].

For more efficient procedure to identify chickpea resistant lines in the available germplasm against *Fusarium* wilt disease the molecular markers can be used for chickpea screening to facilitate gene pyramiding and molecular breeding [44]. The previous workers [45], identified the genetic linkage of resistant genes using different RAPD and SSR markers for various *FOC* races (*FOC* 1, 2, 3, 4 and 5) in inbred chickpea lines developed from resistant and susceptible parental combinations. While, in our study we observed that among molecular markers (5 RAPD and 15 SSR markers) i.e., TA194 at a molecular weight 204 bp showed linkage in chickpea germplasm that was not reported earlier. Thus it was suggested that this SSR primer that successfully separated resistant (1) and susceptible lines with significant linkage to allele for resistance should be practically utilized for target chickpea breeding resistant to wilt.

The results based on dendrogram, were quite comparable with field observations. Furthermore, the linkage probability of TA194 marker was 85 %. This significant linkage of primer with resistivity against wilt disease was reconfirmed by ROC curve analysis which is recently developed for numerous agricultural applications to evaluate the performance of diagnostic experiments in the form of graphical representation [46–49].

Furthermore, in present study the coefficient of correlation of the marker TA194 with disease resistant gene (*FOC* locus), Factor 1 was highly significant at *P* ≥ 0.01. Thus the SSR marker has shown strong association with presence of allele for resistance. The PCR amplification using TA194 for certain accessions scored multiple bands, reported in earlier studies [50]. Therefore, re-synthesis of valid SSR markers is required with single amplified locus. One of the reasons of the appearance of multiple bands is the presence of cryptic sites of the primer binding sites [51]. The accessions 2273 (R) and 3058 (MR) did not show any sort of band during PCR amplification that may be due to mutation in primer binding site or absence of the locus, because these accessions were found resistant during field screening.

The evaluation and selection of superior genotypes using various scientific techniques for utilization of yield enhancement on the basis of performance stability is considered an important research study all over the world. For which the initial step is to control the devastating *Fusarium* wilt disease of the crop through MAS to develop disease resistant germplasm of cultivated chickpea in Pakistan. The present study however selected wilt resistant genotypes using SSR marker TA194 that can provide an opportunity in marker assisted breeding for yield improvement of the crop.
